# Predictive value of insulin resistance metabolic score for cardiovascular disease in Chinese arthritis patients: a prospective cohort study

**DOI:** 10.1093/rheumatology/keaf048

**Published:** 2025-01-25

**Authors:** Wen-kai Ke, Ling-ling Xu, Ni Luo

**Affiliations:** Department of Gerontology, CR & WISCO General Hospital, Wuhan University of Science and Technology, Wuhan, Hubei, China; Wuhan University of Science and Technology, Wuhan, Hubei, China; Department of Gerontology, CR & WISCO General Hospital, Wuhan University of Science and Technology, Wuhan, Hubei, China; Department of Gerontology, CR & WISCO General Hospital, Wuhan University of Science and Technology, Wuhan, Hubei, China

**Keywords:** METS-IR, arthritis, cardiovascular disease, prospective cohort study, dose–response relationship

## Abstract

**Objectives:**

Although patients with arthritis have significantly increased cardiovascular disease (CVD) risk, effective prediction tools remain limited. This study aimed to evaluate the predictive value of the Metabolic Score for Insulin Resistance (METS-IR) for CVD events among Chinese patients with arthritis.

**Methods:**

Using data from the China Health and Retirement Longitudinal Study (CHARLS), we conducted a 7-year prospective cohort study (2011–2018) involving 1059 patients with arthritis. The primary exposure was baseline METS-IR, and the primary outcome was incident CVD. Multivariate Cox regression models were used to analyse the association between METS-IR and CVD risk, adjusting for demographic characteristics and lifestyle factors.

**Results:**

After adjusting for confounding factors, each quartile increase in METS-IR was associated with a 36% increased risk of CVD (hazard ratio [HR] = 1.36, 95% CI: 1.14–1.61, *P* < 0.001). Compared with the lowest quartile, the highest quartile showed a 63% increased risk (HR = 1.63, 95% CI: 1.12–2.37, *P* < 0.05), demonstrating a significant dose–response relationship (*P* for trend < 0.05).

**Conclusion:**

METS-IR serves as an effective tool for predicting CVD risk among Chinese patients with arthritis, providing new strategies for early risk identification and prevention.

## Graphical Abstract

**Figure:**
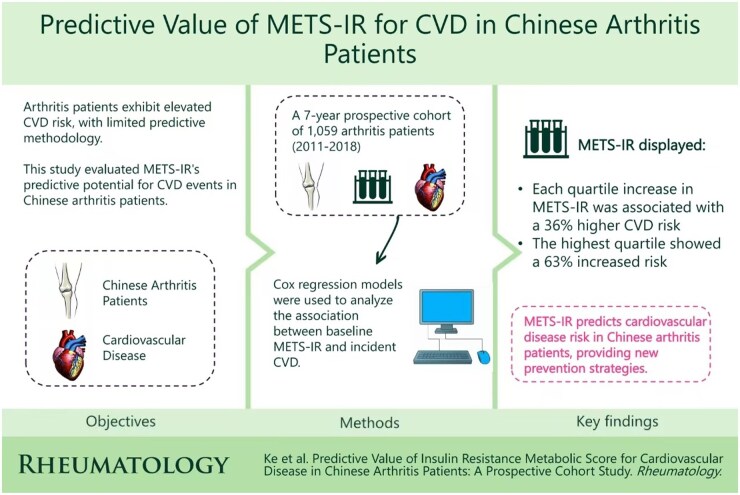

Rheumatology key messages

- Seven-year prospective study reveals METS-IR’s potential in predicting cardiovascular risk for arthritis patients.
- Each METS-IR quartile increase associates with 36% higher cardiovascular risk in arthritis patients.
- Study offers innovative systematic approach for early cardiovascular risk identification in arthritis patients.

## Introduction

Arthritis, encompassing various forms such as osteoarthritis and rheumatoid arthritis, represents a prevalent chronic condition worldwide. Global epidemiological data indicate that the prevalence of osteoarthritis is approximately 7.6% [1], while rheumatoid arthritis affects about 0.46% of the population [2]. In China, according to the China Health and Retirement Longitudinal Study (CHARLS), the overall prevalence of arthritis among individuals aged 45 years and above reaches 31.4% (95% CI: 30.3–32.4%) [3]. Epidemiological evidence has demonstrated a significant association between arthritis and increased cardiovascular risk. A meta-analysis revealed that patients with rheumatoid arthritis exhibit substantially higher risks of cardiovascular events (relative risk 1.48, 95% CI: 1.36–1.62) compared with the general population [4].

Insulin resistance has been established as one of the key pathophysiological mechanisms underlying cardiovascular disease (CVD) development [5, 6]. The Metabolic Score for Insulin Resistance (METS-IR), which integrates multiple metabolic parameters including triglycerides (TG), fasting glucose and BMI, is calculated using the formula: Ln((2 × fasting glucose + triglycerides) × BMI/Ln(HDL-C) [7, 8]. This scoring system has demonstrated significant associations with metabolic syndrome and type 2 diabetes risk. In a study involving 6144 participants, METS-IR showed robust diagnostic performance for metabolic syndrome prediction [9]. Recent evidence has revealed a strong association between elevated METS-IR levels and cardiovascular mortality risk in patients with rheumatoid arthritis (HR = 4.59, 95% CI: 1.98–10.67) [10].

While previous studies have demonstrated a positive association between METS-IR and cardiovascular risk in arthritis patients, several important limitations in the existing literature warrant attention. First, research has predominantly focused on rheumatoid arthritis patients, with limited evaluation of other arthritis subtypes, such as osteoarthritis [11]. Second, most studies employed cross-sectional or short-term follow-up designs, precluding the establishment of causal relationships [12]. Third, there is a notable absence of large-scale prospective cohort data specifically addressing the Chinese population [13]. These limitations have constrained the clinical application of METS-IR as a cardiovascular risk prediction tool in arthritis patients.

To address these limitations, we conducted a prospective cohort study based on the CHARLS database to investigate the association between baseline METS-IR and CVD risk over a seven-year follow-up period. This study offers several notable strengths: it represents the first long-term follow-up study in Chinese arthritis patients, utilizes nationally representative large-scale data, employs standardized assessment tools and accounts for multiple potential confounding factors including age, gender and lifestyle characteristics [14]. The findings from this study will provide a novel predictive tool for early identification of cardiovascular risk in arthritis patients and establish an evidence base for developing targeted prevention strategies.

## Method

### Study population

This prospective cohort study utilized data from the China Health and Retirement Longitudinal Study (CHARLS). CHARLS employed a multistage stratified probability sampling method, with baseline surveys conducted between July 2011 and June 2012, and follow-up extending through December 2018, yielding a seven-year follow-up period. The study covered 150 county-level units and 450 village-level units across mainland China (excluding Tibet, Qinghai and Xinjiang), encompassing ∼10 000 households.

Inclusion criteria comprised: (1) age ≥45 years and (2) confirmed arthritis diagnosis. Exclusion criteria included: (1) pre-existing CVD at baseline, (2) current hypoglycemic or lipid-lowering therapy and (3) missing data for key variables. All investigators underwent standardized training and followed uniform data collection protocols.

**Figure 1.**
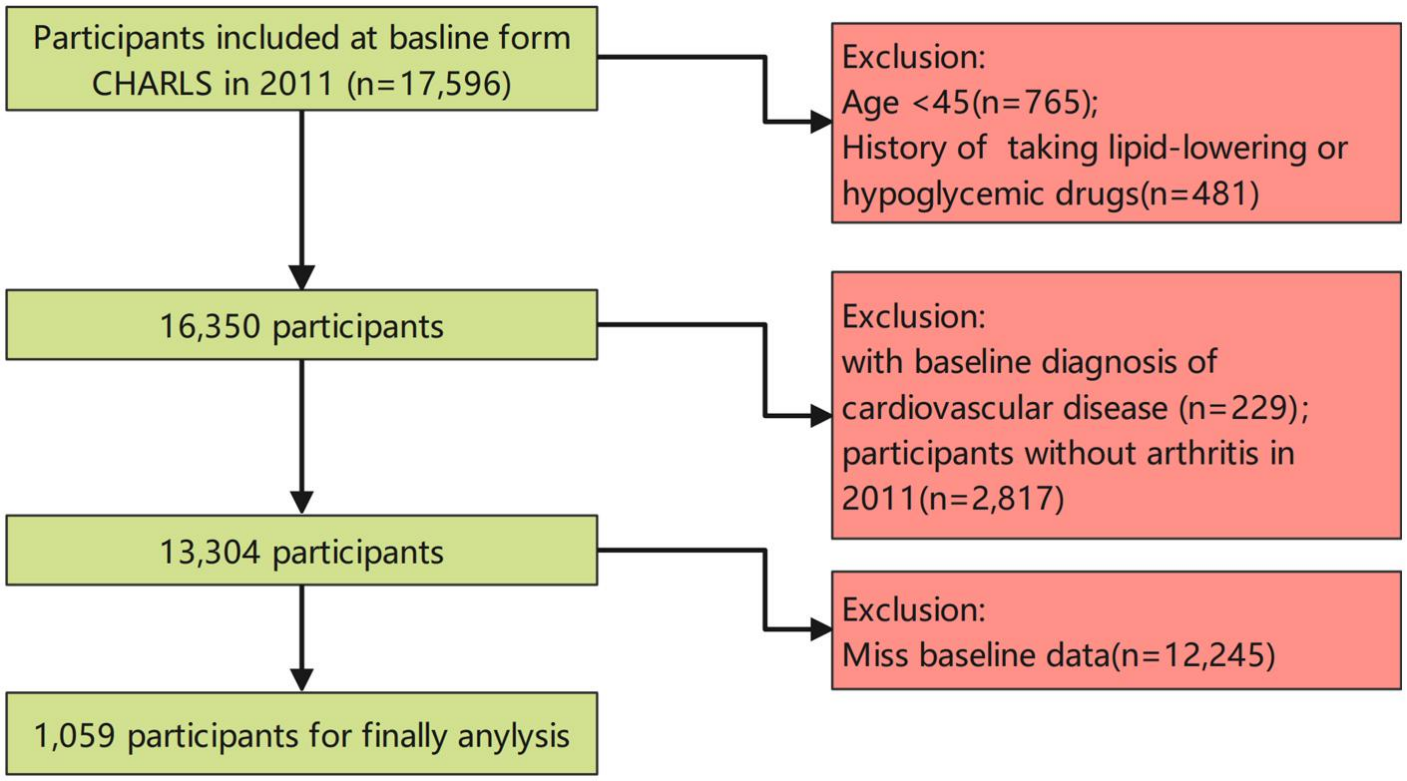
Flow chart for the selection of participants in the cohort study

Of the 17 596 participants who completed both physical examinations and questionnaire assessments at the 2011 baseline survey, 16 537 were subsequently excluded based on the following criteria: age below 45 years (*n* = 765), history of lipid-lowering or hypoglycemic medication use (*n* = 481), incomplete baseline data (*n* = 12 245), absence of arthritis at 2011 baseline (*n* = 2817) and baseline CVD diagnosis (*n* = 229). The final analytical cohort comprised 1059 eligible participants. Figure 1 presents a detailed flowchart of the participant selection process.

### Variables

The exposure variable was the METS-IR. Blood samples were collected at baseline after participants fasted for 8 h, with venous blood drawn by qualified healthcare professionals. All biomedical procedures were performed by certified personnel following standardized protocols. The specimens were maintained at an optimal temperature of 4°C and immediately transported to the central laboratory (You'anmen Clinical Laboratory Center, Capital Medical University, Beijing) for advanced diagnostic assessment. Enzymatic colorimetric methods were used to determine precise measurements of glucose concentration, TG, and high-density lipoprotein cholesterol (HDL-C). METS-IR was calculated using the formula: Ln((2 × fasting glucose[mg/dl] + triglycerides[mg/dl]) × BMI[kg/m^2^]/ln(HDL-C[mg/dl]).

Covariates included: (1) demographic characteristics: age (years), sex (male/female), marital status (married/unmarried), education level (primary school or below/high school or above), residential area (urban/rural); (2)lifestyle factors: smoking status (never/current/former), alcohol consumption (never/more than once per month/less than once per month), sleep duration (h/day); (3) clinical indicators: body mass index (kg/m^2^), waist circumference (cm), systolic blood pressure (mmHg), diastolic blood pressure (mmHg). Covariate selection was based on previous research and clinical expert consensus, considering their potential influence on insulin resistance and CVD.

### Arthritis and CVD diagnosis

Information on arthritis was based on self-reports; when the interviewer asked: ‘Have you been diagnosed with arthritis by a doctor?’ and the respondents answered ‘Yes’, they were classified as arthritis patients [3]. The primary end point of this study was the incidence of CVD during the follow-up period (from 2013 to 2018). In alignment with previous related research [15], information regarding the diagnosis of CVD was collected using a standardized question: ‘Have you been diagnosed with [heart attack, coronary heart disease, angina, congestive heart failure, stroke, or other heart problems] by a doctor?’ The CHARLS study team implemented rigorous quality control measures for data recording and verification to ensure the reliability of the data [14].

### Ethics statement

This study was approved by the Biomedical Ethics Committee of Peking University (approval number: IRB00001052-11014). As a prospective study, written informed consent was obtained from all participants. The research was conducted in strict accordance with the Declaration of Helsinki and the Ethical Review Measures for Biomedical Research Involving Humans. All data were de-identified, and participant privacy was rigorously protected. Study data were stored on encrypted servers with access restricted to authorized research personnel only. Participants retained the right to withdraw from the study at any time without compromising their access to routine medical care.

### Statistical analysis

Continuous variables were expressed as mean ± standard deviation and compared between CVD and non-CVD groups using independent sample *t*-tests. Categorical variables were presented as numbers (percentages) and compared using χ^2^ tests. Multivariate Cox regression models were employed to evaluate the association between METS-IR and CVD risk. Three sequential models were constructed with progressive adjustment for confounding factors: Model 1 was unadjusted; Model 2 adjusted for age, sex, education level, residential area and marital status; Model 3 further adjusted for smoking status, alcohol consumption, sleep duration and blood pressure. Results were presented as HR with 95% CIs. Stratified analyses were conducted to assess the consistency of associations across subgroups (sex, residential area, alcohol consumption patterns and sleep duration). Interaction effects between METS-IR and these stratification variables were tested. To evaluate dose–response relationships, METS-IR values were categorized into quartiles, with the lowest quartile serving as the reference group. Tests for linear trend were performed. Restricted cubic spline (RCS) analysis was conducted to examine potential non-linear relationships between METS-IR and CVD risk (computing *P*-values for overall association and non-linearity). Kaplan–Meier curves and the log-rank test were used to compare the cumulative risk of CVD among four subgroups.

Statistical analyses were performed using R version 4.4.2, RCS analysis was performed using the ‘rms’ package, and survival analysis was performed using the ‘survival’ package. Statistical significance was defined as two-tailed *P*-values < 0.05.

## Results

### Baseline characteristics

Table 1 presents the baseline characteristics of study participants. The study included 1059 subjects, comprising 243 participants in the CVD group and 816 in the non-CVD group. Significant differences were observed between the groups across multiple demographic and clinical parameters. Participants in the CVD group were significantly older than those in the non-CVD group (60.4 ± 8.0 *vs* 58.0 ± 8.3 years, *P* < 0.001) and had a higher proportion of unmarried individuals (14.8% *vs* 9.6%, *P* = 0.028). Regarding anthropometric measurements, the CVD group demonstrated significantly higher body mass index (24.5 ± 4.0 *vs* 23.5 ± 3.5 kg/m^2^, *P* < 0.001) and waist circumference (87.1 ± 14.7 *vs* 83.8 ± 12.2 cm, *P* < 0.001).Clinical parameters showed that the CVD group had significantly elevated fasting glucose levels (109.6 ± 30.7 *vs* 106.1 ± 21.9 mg/dl, *P* = 0.045) and systolic blood pressure (132.1 ± 23.0 *vs* 127.4 ± 19.7 mmHg, *P* = 0.002). Notably, the CVD group exhibited significantly higher METS-IR values (37.0 ± 8.3 *vs* 35.1 ± 7.2, *P* < 0.001) and corresponding interquartile range increments (3.7 ± 0.8 *vs* 3.5 ± 0.7, *P* < 0.001). No significant differences were observed between groups in lipid profiles, CRP levels or lifestyle factors such as smoking and alcohol consumption.

**Table 1.** Demographic and clinical characteristics of participants stratified by cardiovascular disease status

Characteristics	Overall (*n* = 1059)	Non-CVD (*n* = 816)	CVD (*n* = 243)	*P*-value
**Demographic characteristics**				
Age, years	58.6 ± 8.3	58.0 ± 8.3	60.4 ± 8.0	<0.001
Female, *n* (%)	662 (62.5)	499 (61.2)	163 (67.1)	0.110
Marital status, *n* (%)				
Married	945 (89.2)	738 (90.4)	207 (85.2)	0.028
Non-married	114 (10.8)	78 (9.6)	36 (14.8)	
Education level, *n* (%)				
High school or above	292 (27.6)	224 (27.5)	68 (28.0)	0.935
Primary school or below	767 (72.4)	592 (72.5)	175 (72.0)	
**Clinical measurements**				
BMI, kg/m²	23.7 ± 3.6	23.5 ± 3.5	24.5 ± 4.0	<0.001
Waist circumference, cm	84.6 ± 12.9	83.8 ± 12.2	87.1 ± 14.7	<0.001
SBP, mmHg	128.5 ± 20.6	127.4 ± 19.7	132.1 ± 23.0	0.002
DBP, mmHg	74.2 ± 11.4	73.9 ± 11.2	75.3 ± 12.1	0.088
**Laboratory parameters**				
Fasting glucose, mg/dL	106.9 ± 24.3	106.1 ± 21.9	109.6 ± 30.7	0.045
Triglycerides, mg/dL	127.9 ± 84.4	126.7 ± 84.7	131.8 ± 83.5	0.409
HDL-C, mg/dL	52.5 ± 15.1	52.7 ± 14.9	52.0 ± 15.6	0.519
LDL-C, mg/dL	118.7 ± 34.3	117.9 ± 34.1	121.6 ± 35.0	0.134
CRP, mg/L	2.8 ± 7.4	2.6 ± 6.9	3.3 ± 8.9	0.207
METS-IR	35.5 ± 7.5	35.1 ± 7.2	37.0 ± 8.3	<0.001
METS-IR per IQR	3.6 ± 0.7	3.5 ± 0.7	3.7 ± 0.8	<0.001
**Lifestyle factors**				
Diabetes mellitus, *n* (%)	52 (4.91)	37 (4.53)	15 (6.17)	0.299
Sleep time, h	6.1 ± 1.9	6.1 ± 1.9	6.1 ± 2.1	0.921

Data are presented as mean ± standard deviation or *n* (%). *P*-values were calculated using Student’s *t*-test for continuous variables and χ^2^ test for categorical variables. *P* < 0.05 was considered statistically significant.

CVD: cardiovascular disease; SBP: systolic blood pressure; DBP: diastolic blood pressure; HDL-C: high-density lipoprotein cholesterol; LDL-C: low-density lipoprotein cholesterol; METS-IR: Metabolic Score for Insulin Resistance; IQR: interquartile range.

### Dose–Response relationship between METS-IR and CVD

Table 2 demonstrates the relationship between METS-IR and incident CVD risk among arthritis patients. The METS-IR showed a significant association with CVD risk. After adjusting for potential confounders including age, sex, education level, residential area, marital status, smoking status, alcohol consumption, sleep duration and blood pressure (Model 3), each interquartile increase in METS-IR was associated with a 36% higher risk of CVD (HR = 1.36, 95% CI: 1.14–1.61, *P* < 0.001).

**Table 2.** Association between METS-IR and cardiovascular disease risk

Variables	Model 1	Model 2	Model 3
METS-IR (per IQR)	1.34 (1.14–1.57)***	1.37 (1.17–1.62)***	1.36 (1.14–1.61)***
METS-IR quartiles			
Q1	1.00 (Reference)	1.00 (Reference)	1.00 (Reference)
Q2	1.05 (0.72–1.54)	1.07 (0.73–1.57)	1.10 (0.75–1.62)
Q3	1.20 (0.83–1.73)	1.24 (0.85–1.81)	1.20 (0.82–1.77)
Q4	1.56 (1.10–2.23)*	1.64 (1.14–2.36)**	1.63 (1.12–2.37)*
P for trend	1.03 (1.01–1.05)**	1.03 (1.01–1.05)**	1.03 (1.01–1.05)*

Data are presented as hazard ratio (95% CI).

Model 1: Unadjusted.

Model 2: Adjusted for age, gender, education level, location and marital status.

Model 3: Adjusted for Model 2 covariates plus smoking status, drinking status, sleep time, SBP and DBP.

* *P* < 0.05,

** *P* < 0.01,

*** *P* < 0.001.

METS-IR: metabolic score of insulin resistance; IQR: interquartile range; SBP: systolic blood pressure; DBP: diastolic blood pressure.

When participants were stratified by METS-IR quartiles, those in the highest quartile demonstrated a significant 63% increased risk of CVD compared with those in the lowest quartile (HR = 1.63, 95%CI: 1.12–2.37, *P* < 0.05). Trend analysis revealed a significant dose–response relationship between METS-IR levels and CVD risk (*P* for trend < 0.05). Notably, this association remained stable before and after adjustment for potential confounding factors, suggesting that METS-IR may serve as an independent predictor of CVD risk.

**Figure 2.**
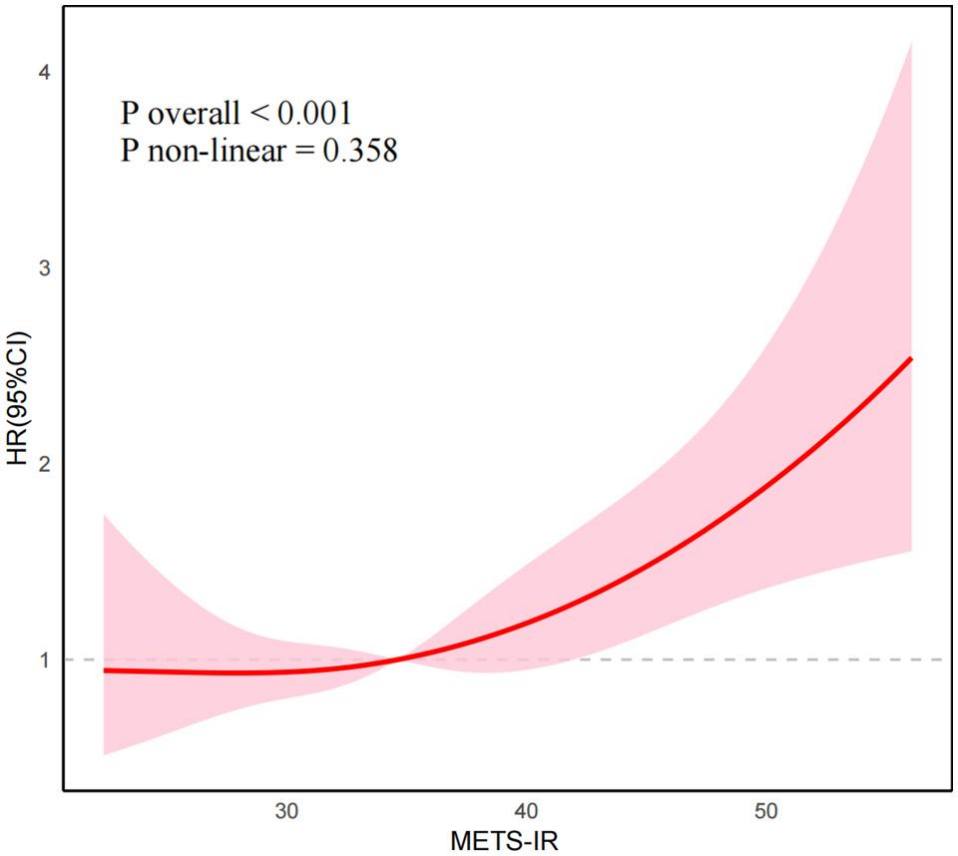
Restricted cubic spline of the association between METS-IR and the risk of CVD

RCS analysis, as illustrated in Fig. 2, demonstrated the dose–response relationship between METS-IR and incident CVD risk in arthritis patients. The results indicated a linear dose–response association between METS-IR and incident CVD risk (*P* overall <0.001, *P* non-linear =0.358).

### Kaplan–Meier survival curve analysis

**Figure 3.**
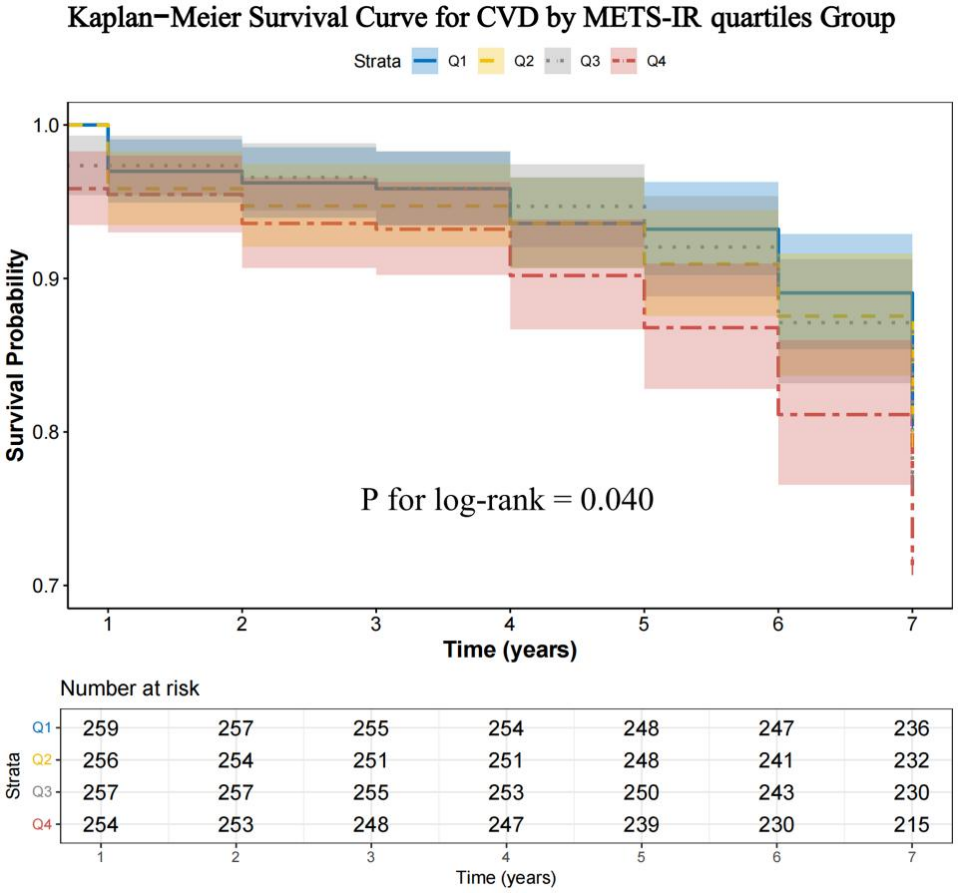
Kaplan–Meier Survival Curve for CVD by METS-IR quartiles group

In the analysis of Kaplan–Meier survival curves for arthritis patients, it was observed that the probability of survival significantly decreased in patients with CVD as the METS-IR quartile increased (Fig. 3). The log-rank test revealed a statistically significant difference in survival rates across different METS-IR quartiles (*P* = 0.040), suggesting that individuals with higher levels of insulin resistance are at an elevated risk of CVD.

### Stratified analysis

**Figure 4.**
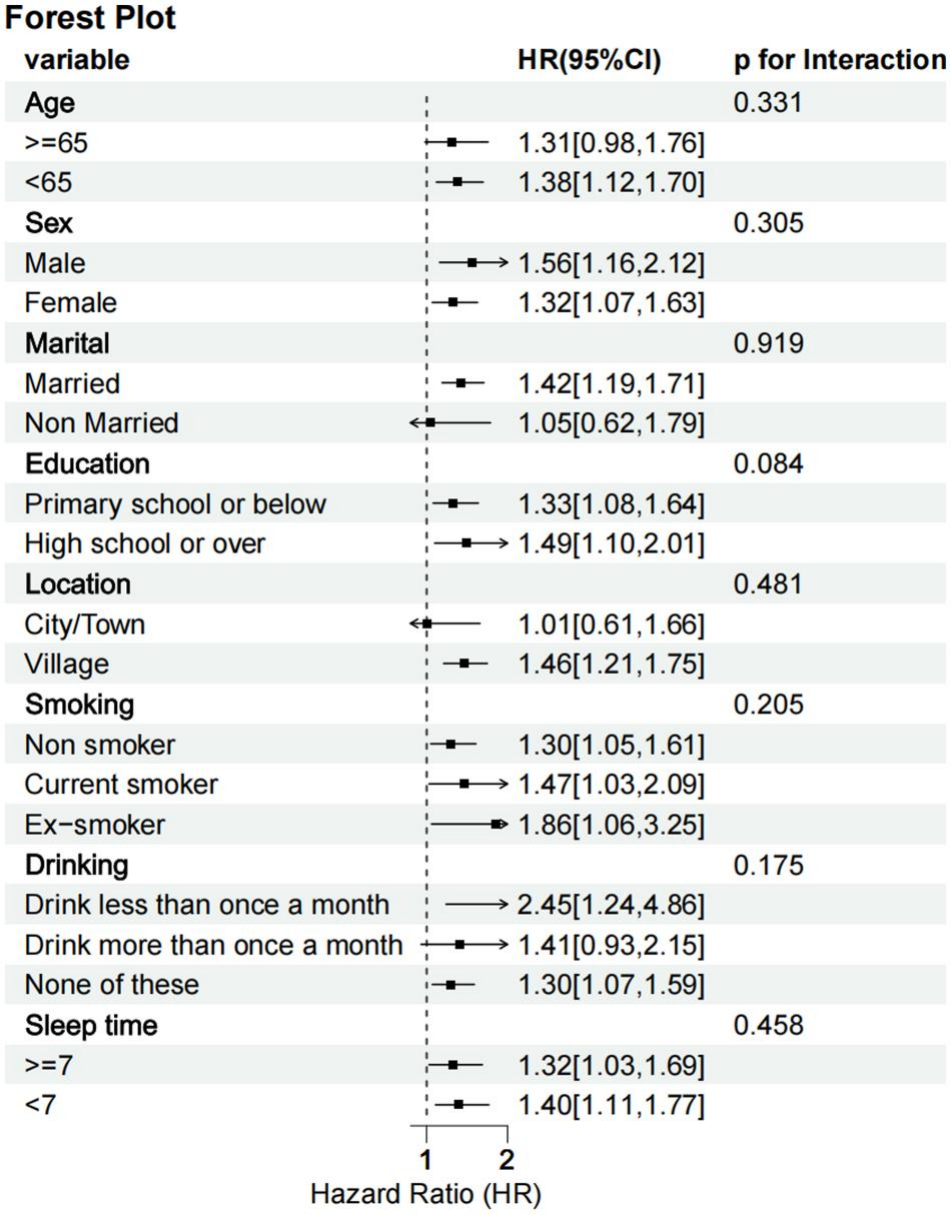
The associations between Mets-IR and incident CVD differs among subgroups

Consistent associations were observed across demographic and lifestyle subgroups, with no significant interaction effects detected (all interaction *P*-values >0.05), as shown in Fig. 4. The strongest associations were observed in alcohol consumption patterns, with an HR of 2.45 (95% CI: 1.24–4.86) for individuals consuming alcohol less than once monthly and 1.30 (95% CI: 1.07–1.59) for alcohol abstainers. Subgroup analyses revealed consistent associations across various demographic and lifestyle characteristics.

The absence of significant interaction effects suggests that the observed associations were independent of demographic characteristics and lifestyle factors.

## Discussion

This study represents the first systematic investigation of the association between the METS-IR and CVD risk using a 7-year follow-up data from 1059 Chinese arthritis patients in the CHARLS database. This prospective cohort study utilized nationally representative data and accounted for multiple confounding factors. After adjusting for demographic characteristics and lifestyle factors, we found that each interquartile increase in METS-IR was associated with a 36% higher risk of CVD (HR = 1.36, 95% CI: 1.14–1.61, *P* < 0.001). Notably, this association demonstrated a significant dose–response relationship (*P* for trend < 0.05). In the Kaplan–Meier survival curve analysis, arthritis patients in the higher METS-IR quartile exhibited a significantly lower probability of survival. The log-rank test (*P* = 0.040) confirmed that this difference was statistically significant.

To better understand the mechanistic basis of our findings, we explored the potential biological links between METS-IR and CVD risk in arthritis patients. The observed association appears to be mediated through metabolic-inflammatory cascade reactions: insulin resistance leads to adipose tissue dysfunction, resulting in increased secretion of pro-inflammatory factors (TNF-α, IL-6, etc.), which not only exacerbates the inflammatory state of arthritis but also promotes atherosclerosis development [16, 17]. Furthermore, insulin resistance-induced endothelial dysfunction manifests as reduced NO bioavailability and impaired vasodilation, while concurrent metabolic disorders elevate oxidative stress levels, collectively accelerating atherosclerosis progression [18, 19].

Previous studies provide important supporting evidence. Zhou and Gao [10] demonstrated a significant association between elevated METS-IR levels and cardiovascular mortality risk in a retrospective cohort study of 1218 rheumatoid arthritis patients. Our study extends these findings through long-term prospective follow-up, examining the association between METS-IR and cardiovascular events. We observed a statistically significant effect (HR = 1.36), which suggests that this association holds substantial clinical importance. Bello-Chavolla *et al*. [7] validated the metabolic score’s accuracy in predicting visceral fat content and cardiometabolic status, while our study extends its application to arthritis populations.

Notably, Crowson *et al*. [12] revealed in their multicentre study that traditional cardiovascular risk factors exhibit unique patterns of influence in rheumatoid arthritis patients, highlighting the necessity for developing targeted risk assessment tools. Solomon *et al*. [5] reported that disease activity closely correlates with cardiovascular event risk, corroborating findings from England *et al*. [20] regarding cumulative cardiovascular complication risk in rheumatoid arthritis patients. This association likely stems from insulin resistance-induced chronic inflammation and endothelial dysfunction.

Our findings have important clinical and translational implications. First, METS-IR, as an integrated metabolic scoring tool incorporating multiple metabolic parameters, is simple to implement and cost-effective, making it suitable for widespread adoption in primary healthcare settings [21]. Second, the demonstrated dose–response relationship between METS-IR and cardiovascular risk provides clinicians with an objective, quantifiable risk assessment tool [22]. The robust predictive performance across various population subgroups indicates excellent generalizability of this scoring system.

Based on these findings, we recommend incorporating METS-IR into routine cardiovascular risk screening protocols for arthritis patients. Early interventions, including enhanced lifestyle management and metabolic parameter monitoring, should be considered for patients with elevated METS-IR scores. Furthermore, our study, based on large-scale prospective data from the Chinese population, provides crucial evidence for developing localized prevention strategies. Future research should explore the combined utility of METS-IR with other risk prediction models and investigate potential variations in predictive performance across different types of arthritis.

Our study leverages the CHARLS national prospective cohort database with multi-stage stratified sampling to ensure population representativeness. The analytical approach included progressive adjustment models, stratified analyses and RCS analysis to examine METS-IR-cardiovascular relationships comprehensively. Rigorous outcome verification protocols and strict adherence to ethical guidelines further enhanced study reliability.

### Limitations

Despite providing important clinical evidence, several limitations of our study warrant consideration. First, due to the absence of a comprehensive arthritis classification system in the database utilized, we were unable to ascertain the precise implications of these findings for specific types of arthritis, such as osteoarthritis, rheumatoid arthritis and spondyloarthritis.

Second, the study’s generalizability is limited by its focus on Chinese arthritis patients aged 45 years and above. Additionally, as an observational study, causal relationships cannot be established, and unmeasured confounding factors may exist.

### Future directions

To address these limitations, future research should conduct longer-term follow-up studies (>10 years), expand population coverage across different age groups and ethnicities, and perform interventional studies. Additionally, investigating interactions with disease activity, developing integrated prediction models incorporating METS-IR, and evaluating its utility across different types of arthritis would further enhance our understanding of this risk assessment tool.

## Conclusion

METS-IR serves as an effective tool for predicting CVD risk among Chinese patients with arthritis, providing new strategies for early risk identification and prevention.

## Data Availability

The data used in this study were derived from the China Health and Retirement Longitudinal Study (CHARLS), which is publicly available at http://charls.pku.edu.cn/. Researchers can obtain these data by registering on the CHARLS website and signing a data use agreement.
